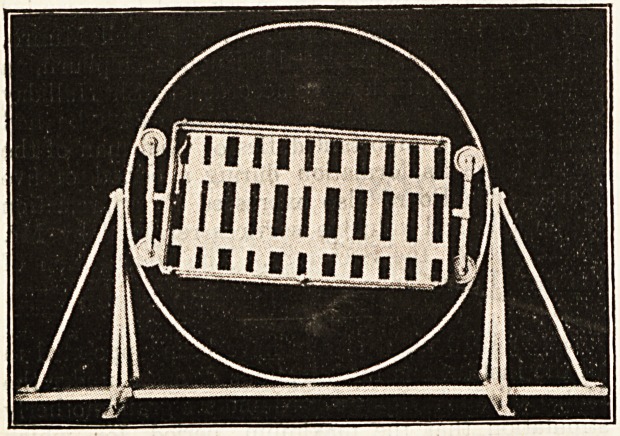# The Utility Bed

**Published:** 1921-02-05

**Authors:** 


### The Utility Bed.

We are able to give an illustration of the ingenious "Utility Bed," produced by the Gloucestershire Surgical Appliances Co., of Cheltenham. By its means a patient can be comfortably held in any position, even upside down, and this wide facility will suggest a large number of useful nursing purposes to which the bed may be put.

A locking device is provided to enable the bed to be fixed firmly in any position the doctor or nurse requires the patient to take.

The mattress is double in order that when the patient is lying in the face-downward position the body is still resting on the mattress, which is held in position by wide strips of webbing.

It is claimed that the principle feature of the bed is that it is invaluable in hospitals, nursing homes, and other institutions for use in treatment of disease or accident where non-movement of the patient is essential, for instance, in the case of a broken femur.

The bed has longitudinal and lateral movements. The mattress is made up of six separate sections, each of which can be easily removed as required, thus providing facilities for dressing, etc., any part of the patient's body.

## Figures and Tables

**Figure f1:**